# Accelerating Families of *Fuzzy K-Means* Algorithms for Vector Quantization Codebook Design

**DOI:** 10.3390/s16111963

**Published:** 2016-11-23

**Authors:** Edson Mata, Silvio Bandeira, Paulo de Mattos Neto, Waslon Lopes, Francisco Madeiro

**Affiliations:** 1 Center of Science and Technology, Catholic University of Pernambuco (UNICAP), Recife 50050-900, Brazil; edsonmata@hotmail.com (E.M.); silvio@c3.unicap.br (S.B.); madeiro@c3.unicap.br (F.M.); 2 Centro de Informática, Universidade Federal de Pernambuco (UFPE), Recife 50740-560, Brazil; psgmn@cin.ufpe.br; 3 Department of Electrical Engineering, Center of Alternative and Renewable Energy, Federal University of Paraíba (UFPB), João Pessoa 58038-130, Brazil

**Keywords:** *fuzzy K-means*, vector quantization, computational complexity

## Abstract

The performance of signal processing systems based on vector quantization depends on codebook design. In the image compression scenario, the quality of the reconstructed images depends on the codebooks used. In this paper, alternatives are proposed for accelerating families of *fuzzy K-means* algorithms for codebook design. The acceleration is obtained by reducing the number of iterations of the algorithms and applying efficient nearest neighbor search techniques. Simulation results concerning image vector quantization have shown that the acceleration obtained so far does not decrease the quality of the reconstructed images. Codebook design time savings up to about 40% are obtained by the accelerated versions with respect to the original versions of the algorithms.

## 1. Introduction

Signal compression techniques aim at decreasing the number of bits needed to represent the signal (such as speech, image, audio and video), enhancing the efficiency both of transmission and storage. Compression techniques are widely used in applications with storage and bandwidth constraints, such as: storage of medical images, satellite transmissions, voice communication in mobile telephony and videoconference. One of the many techniques used to achieve signal compression is vector quantization (VQ), in which a codebook is used for signal reconstruction.

Vector quantization [1,2] is a lossy compression technique, which uses a mapping *Q* of a vector *X*, in a *K*-dimensional Euclidean space, into another vector belonging to a finite subset *W* of ${\mathbb{R}}^{K}$:

(1) $$Q:{\mathbb{R}}^{K}\to W.$$

The finite subset *W* is called a *codebook*. Each codebook element $w_{j}$, 1≤j≤N, is called a *codevector*. The number of components in the codevectors is the *dimension* (*K*). The size of the codebook is the number of codevectors, denoted by *N*. In several speech coding [3,4,5] and image coding [6,7,8,9] systems, VQ has been used successfully, leading to high compression rates. VQ has also been used in other applications, such as speaker identification [10,11], information security such as steganography and digital watermarking [12,13,14,15,16,17,18], and classification of pathological voice signals [19].

Vector quantization is an extension of scalar quantization in a multidimensional space. The performance of VQ depends on the designed codebooks. The prevailing algorithm for codebook design is Linde-Buzo-Gray (LBG) [20], also known as *Generalized Lloyd Algorithm* (GLA) or *K*-*means*. Other examples of codebook design algorithms are: *fuzzy* [7,21,22], competitive learning [23], memetic [24], genetic [25], firefly [26] and honey bee mating optimization [27].

In vector quantization of a digital image, a codebook of size *N* is used, consisting in *K*-dimensional vectors. The process replaces blocks of pixels from the corresponding image by the most similar blocks of pixels in the codebook. So, the better the codebook, the higher the quantized image quality.

Typical grouping approaches used in VQ split in two categories: *crisp* and *fuzzy* clustering. Traditionally, crisp clustering is executed by the *K*-*means* algorithm. Due to initialization dependency, *K*-*means* can be stuck in undesired local minima. On the other hand, fuzzy clustering is usually performed by the *fuzzy K*-*means* (FKM) algorithm [28]. FKM attributes each training pattern to every other cluster with different pertinence degrees [29]. Therefore, FKM is able to reduce the random initialization dependency [7,29,30,31] at a high computational cost.

The *K*-*means* (KM) and *fuzzy* clustering algorithms, e.g., *fuzzy K*-*means* (FKM), have been used in a wide range of scenarios and applications, such as: digital soil pattern recognition [32], archaeology [33], indoor localization [34], discrimination of cabernet sauvignon grapevine elements [35], white blood cell segmentation [36], abnormal lung sounds diagnosis [37], intelligent sensor networks in agriculture [38], magnetic resonance image (MRI) segmentation [39,40], speaker recognition [41] and image compression by VQ [29,42,43].

The aforementioned works show that clustering algorithm applications include image coding, biometric authentication, pattern recognition, among others. The performance evaluation of the clustering algorithms depends on the application. In signal compression, an important aspect is the quality of the reconstructed signal. In pattern recognition systems, an important figure of merit is the recognition rate. The processing time of the clustering algorithms is also a relevant aspect. In this paper, techniques are presented for accelerating families of *fuzzy K*-*means* algorithms applied to VQ codebook design for image compression. Simulations show that the presented techniques lead to a decrease in processing time for codebook design, while preserving its overall quality.

One of the many techniques used in this work is the *Equal*-*average Nearest Neighbor Search* (ENNS) [44,45], which is usually used in the minimum distance coding phase of VQ. However, in this paper, ENNS is used in some of *fuzzy K*-*means* families, precisely in the partitioning of the training set. The acceleration of FKM algorithms is also obtained by the use of a lookahead approach in the crisp phase of such algorithms, leading to a decrease in the number of iterations.

The remaining sections are organized as follows: Section 2 covers *K*-*means algorithm* and *fuzzy K*-*means* families. Section 3 presents modified versions of *fuzzy K*-*means* families. In Section 4, nearest neighbor search techniques are introduced with focus in the scenario of accelerating codebook design. The results and final considerations are presented in Section 5 and Section 6, respectively.

## 2. Codebook Desing Techniques 

Vector quantization performance is highly dependent on codebook quality. The codebook is a set of reference patterns or *templates*. In digital image coding, the codebook corresponds to a set of reference blocks of pixels. In this paper, *K*-*means* algorithm and *fuzzy K*-*means* families are the techniques under consideration for codebook design.

The main difference between *K*-*means* and *fuzzy K*-*means* algorithms is that, in the former, each training vector belongs to one quantization cell. In the latter, each training vector can be associated to more than one quantization cell, with some degree of pertinence to each cell.

*K*-*means* algorithm partitions the ${\mathbb{R}}^{K}$ vector space by associating each training vector to a single cluster using nearest neighbor search. Therefore, given an input vector $x_{i}$, it belongs to the cluster (cell or Voronoi region):

(2) $$V(w_{j})\;\mathrm{if}\;d\left(x_{i},w_{j}\right)<\;d\left(x_{i},w_{a}\right)\;\forall \;a\neq j,$$

where $d\left(x_{i},w_{j}\right)$ is a distance measure. Euclidean square distance between $x_{i}$ and $w_{j}$ is widely used in digital image vector quantization. In this case, $w_{j}$ is the nearest neighbor (NN) of $x_{i}$, that is,$\;w_{j}$ is the quantized version of $x_{i}$. This is equivalent to $w_{j}=Q\left(x_{i}\right)$. The nearest neighbor search can be associated to a pertinence function:

(3) $$\mu_{j}\left(x_{i}\right)=\;\{\begin{array}{c} \;1,\;\mathrm{if}\;w_{j}=\mathrm{NN}\left(x_{i}\right)\;\\ 0,\;\mathrm{otherwise} \end{array}.$$

The distortion, obtained by representing the training vectors by their corresponding nearest neighbors, is:

(4) $$J_{1}=\sum_{j=1}^{N}\sum_{i=1}^{M}\mu_{j}\left(x_{i}\right)d\left(x_{i},w_{j}\right),$$

in which $x_{i}$ is the *i-*th training vector, 1≤i≤M. As $J_{1}$ is a function of $w_{j}$, in order to minimize the distortion, vectors $w_{j}$ are updated according to:

(5) $$w_{j}=\frac{\sum_{i=1}^{M}\mu_{j}\left(x_{i}\right)x_{i}}{\sum_{i=1}^{M}\mu_{j}\left(x_{i}\right)},\forall \;j=1,2,\ldots ,N.$$

Equations (2) and (5) are related to the partitioning of the training set and to the codebook update. The algorithm stops at the end of the n-th iteration if:

(6) $$\frac{J_{1}\left(n-1\right)-J_{1}\left(n\right)}{J_{1}\left(n\right)}\leq \;\varepsilon .$$

The input parameters of the *K*-*means* algorithm are: codebook size (*N*), codevectors dimension (*K*) and a distortion threshold *ε* used as stop criterion. 

The *fuzzy K*-*means* algorithm aims at minimizing the distortion between training vectors $x_{i}$ and codevectors $w_{j}$ which compose the codebook. Unlike *K*-*means* algorithm, *fuzzy K*-*means* measures the distortion by [29]:

(7) $$J_{m}=\sum_{j=1}^{N}\sum_{i=1}^{M}\mu_{j}{\left(x_{i}\right)}^{m}d\left(x_{i},w_{j}\right),\;1<m<\infty ,$$

subject to the following conditions:

(8) $$\{\begin{array}{c} \mu_{j}\left(x_{i}\right)\;\epsilon \;\left[0,1\right]\;\forall \;i,\;j,\\ 0<\sum_{i=1}^{M}\mu_{j}\left(x_{i}\right)<M,\\ \sum_{j=1}^{N}\mu_{j}\left(x_{i}\right)=1,\;\forall \;i=1,2,\ldots ,\;M \end{array}$$

As stated in [29], $J_{m}$ function minimization results:

(9) $$\mu_{j}\left(x_{i}\right)=\frac{1}{\sum_{l=1}^{N}{\left(\frac{d\left(x_{i},w_{j}\right)}{d\left(x_{i},w_{l}\right)}\right)}^{\frac{1}{m-1}}}.$$

Therefore, for a given pertinence degree set of functions, the codevectors evolve at each iteration to minimize $\;J_{m}$, according to [29]:

(10) $$w_{j}=\frac{\sum_{i=1}^{M}\mu_{j}{\left(x_{i}\right)}^{m}x_{i}}{\sum_{i=1}^{M}\mu_{j}{\left(x_{i}\right)}^{m}},\forall \;j=1,2,\ldots ,N.$$

The nebulosity at clusters transitions is controlled by parameter m and increases with this parameter.

The input parameters of the FKM algorithm are: the codebook size (*N*), the codevector dimension (*K*), the nebulosity control parameter m ϵ (1,∞), and the distortion threshold ε.

This work uses two *fuzzy K*-*means* families, as proposed in [29]. The development of those algorithms is based on transition from *fuzzy* to crisp mode, being the latter mode equivalent to *K*-*means* algorithm strategy. The algorithm *fuzzy* 1 (FKM1) presents three modifications in its construction when compared to FKM. The first is how the pertinence function is calculated:

(11) $$\mu_{j}\left(x_{i}\right)=f\left(d\left(x_{i},w_{j}\right),\;d_{max}\left(x_{i}\right)\right)={\left(1-\frac{d\left(x_{i},w_{j}\right)}{d_{max}\left(x_{i}\right)}\right)}^{u},$$

in which $d_{max}\left(x_{i}\right)$ gives the maximum distance between the training vectors and codevectors, and u is a positive integer. The second modification concerns the codebook update, defined by Equation (5). The last modification is found in the transition from *fuzzy* to crisp mode. For that purpose, a distortion threshold $\varepsilon^{\prime }$ is defined, with $\varepsilon^{\prime }>\varepsilon$. Therefore, FKM1 algorithm has the following parameters as input: N, K, u and two distortion thresholds—precisely, $\varepsilon^{\prime }$ represents the *fuzzy* to crisp mode transition threshold and ε represents the stop criterion.

The *fuzzy* 2 family (FKM2) uses the same codebook update and pertinence function calculations as proposed by *fuzzy K*-*means* algorithm, that is, Equations (9) and (10), respectively. The only difference is the inclusion of *fuzzy* to crisp mode transition.

## 3. Accelerating *Fuzzy K-Means* Family Algorithm

One of the challenges in the clustering methods is to increase the convergence speed, that is, the decrease in the number of iterations. Some alternatives have been proposed to accelerate *K*-*means* algorithm, as the techniques of Lee et al. [46] and Paliwal-Ramasubramanian [47]. Both techniques recalculate the codevectors at the end of each iteration, according to the expression:

(12) $${w_{j}}^{n+1}={w_{j}}^{n}+s\left(C\left(V\left({w_{j}}^{n}\right)\right)-{w_{j}}^{n}\right),$$

where ${w_{j}}^{n}$ is the codevector at the n-th iteration, s is the scale and $C\left(V\left({w_{j}}^{n}\right)\right)$ is the centroid of the Voronoi region $V\left({w_{j}}^{n}\right)$. Fixed scale s is used in [46]. The modification introduced in [46], proposed in [47], consists in using a scale s which depends on the iteration n, that is:

(13) $$s=1+\frac{v}{v+n}\;,$$

for some v>0.

In this paper, the *fuzzy K*-*Means* families accelerated version uses Equations (12) and (13) in codevectors updating. According to simulation results, for FKM1 and FKM2 algorithms, the scale s leads to savings in the number of iterations when applied to the crisp phase of the algorithms.

## 4. Nearest Neighbor Search Techniques for Accelerating the Codebook Design

When FKM1 and FKM2 algorithms change to crisp mode (which is equivalent to the conventional *K*-*means* algorithm), the complexity of the nearest neighbor search, performed by the *K*-*means*, can be minimized by efficient search techniques. Usually, *K*-*means* algorithm uses *Full Search* (FS) to compute the nearest neighbor, which is highly time consuming. 

A great number of operations can be saved by eliminating poor codevector candidates to the nearest neighbor. This can be accomplished by using search techniques, such as *Partial Distortion Search* (PDS) [48] and *Equal*-*average Nearest Neighbor Search* (ENNS) [44,45]. Both were originally proposed to VQ encoding phase. Instead, in this paper, they are used in FKM1 and FKM2 algorithms. PDS and ENNS apply rejection criteria on codevectors, decreasing, by that means, the time spent in the nearest neighbor search. 

PDS algorithm, as proposed in [48], consists of a traditional technique to computational complexity reduction involved in nearest neighbor search. PDS determines, for any q≤K, if the accumulated distance to the first q codevector components is greater than $d_{min}$ (the minimum distance found in the search so far). If the condition is true, that codevector does not represent the NN. So, it is assumed that the following expression is satisfied:

(14) $$\sum_{l=1}^{q}\;{\left(x_{il}-w_{jl}\right)}^{2}\geq d_{min},$$

where 1≤q≤K, $x_{il}$ is the *l*-th component of training (input) vector $x_{i}$ and $w_{jl}$ is the *l*-th component of codevector $w_{j}$. When this condition is satisfied there is no need to perform the hole calculation for the Euclidean distance between $x_{i}$ and $w_{j}$. With this approach, the number of multiplications, subtractions and additions is reduced, decreasing the search time and, therefore, accelerating the codebook design in comparison to the full search. 

In the ENNS algorithm, the mean for each codevector is calculated and sorted previously. Then, a lookup is performed, using some search algorithm, to find the codevector with mean closest to the mean $m_{x}$ of the current input vector *x*. When such codevector is found, searches do not need to be performed for codevectors whose means $m_{i}$ satisfy the criterion:

(15) $$m_{i}\geq m_{x}+\sqrt{\frac{d_{min}}{K}\;}\text{ or }m_{i}\leq m_{x}-\sqrt{\frac{d_{min}}{K}},$$

where $m_{i}$ is the mean of the *i*-th codevector, $m_{x}$ is the mean of current input vector and $d_{min}$ is the distance between the input vector and the codevector with the nearest mean. 

When the elimination criterion is not satisfied for a given vector, it enters in a waiting list to be looked up later. After all winner candidates to that input vector are collected, a search is performed calculating the square Euclidean distance and the PDS is used. 

ENNS decreases the computational time compared to the full search with *N* (codebook size) memory allocations penalty, compared to PDS. That fact is proved in [49]. Because ENNS was originally used in coding phase, it performs one means sorting, since the codebook vectors were previously designed. However, as for the codebook design, the *K*-*means* algorithm (on the crisp mode of FKM families), at each iteration, updates its codevectors, hereby a new average sorting is needed for each iteration. Acceleration alternatives in the scenario of FKM2 are presented as follows (see Algorithms 1–3). The notation MFKM2 stands for modified *fuzzy K*-*means* family 2, that is, an acceleration (savings in the number of iterations) obtaining by using the scale factor *s* in codebook update.

**Algorithm 1.** Partitioning step of the conventional FKM2 algorithm in crisp modeFor 1≤m≤M
Calculate $d\left(x_{m},w_{j}\right)=\sum_{l=1}^{K}{\left(x_{ml}-w_{jl}\right)}^{2}$, ∀ j=1,2,…,NDetermine the smallest of the N calculated distances. The nearest neighbor of $x_{m}$ is $w_{j}$ such that $d\left(x_{m},w_{j}\right)<d\left(x_{m},w_{o}\right)\;\forall \;o\neq j\;$. In this case, $x_{m}$ is allocated to the Voronoi region $V\left(w_{j}\right)$ Codebook update step of the FKM2 algorithm: Calculate ${w_{j}}^{n}=\frac{\sum_{i=1}^{M}\mu_{j}\left(x_{i}\right)x_{i}}{\sum_{i=1}^{M}\mu_{j}\left(x_{i}\right)},\;\forall \;j=1,2,\ldots ,N$
Codebook update with ${w_{j}}^{n+1}={w_{j}}^{n}$, in which ${w_{j}}^{n}$ is the codevector at the n-th iteration

**Algorithm 2.** Partitioning step of the MFKM2 algorithm in crisp modeFor 1≤m≤M
Calculate $d\left(x_{m},w_{j}\right)=\sum_{l=1}^{K}{\left(x_{ml}-w_{jl}\right)}^{2}$, ∀ j=1,2,…,NDetermine the smallest of the N calculated distances. The nearest neighbor of $x_{m}$ is $w_{j}$ such that $d\left(x_{m},w_{j}\right)<d\left(x_{m},w_{o}\right)\;\forall \;o\neq j\;$. In this case, $x_{m}$ is allocated to the Voronoi region $V\left(w_{j}\right)$ Codebook update step of the MFKM2 algorithm: Calculate ${w_{j}}^{n}=\frac{\sum_{i=1}^{M}\mu_{j}\left(x_{i}\right)x_{i}}{\sum_{i=1}^{M}\mu_{j}\left(x_{i}\right)},\;\forall \;j=1,2,\ldots ,N$
Codebook update with ${w_{j}}^{n+1}={w_{j}}^{n}+s\left(C\left(V\left({w_{j}}^{n}\right)\right)-{w_{j}}^{n}\right)$


It is worth mentioning that other approaches have been proposed in the literature for the purpose of fast codebook search. As an example, the method introduced by Chang and Wu [50] is an interesting partial-search technique based on a graph structure which leads to computational cost savings.

**Algorithm 3.** Partitioning step of the MFKM2 algorithm in crisp mode with the use of ENNS(Calculate *off*-*line* the mean of each input vector) Calculate the mean of each codevector and order the N means in ascending order For 1≤m≤M
Determine the codevector with the minimum absolute difference between its mean and the input vector mean. Obtain $d_{min}$ as the squared Euclidean distance between this codevector and the input vectorEliminate from the search process the codevectors that satisfy: $$m_{i}\geq m_{x}+\sqrt{\frac{d_{min}}{K}}\text{ or }m_{i}\leq m_{x}-\sqrt{\frac{d_{min}}{K}}$$For the remaining codevectors, i.e., those who were not eliminated from the search, apply the PDS algorithm for calculating the distance and update $d_{min}$ (the minimum distance found in the search so far)At the end of the process, the codevector $w_{j}$ corresponding to $d_{min}$ is the nearest neighbor of $x_{m}$. In this case, $x_{m}$ is allocated to the Voronoi region $V\left(w_{j}\right)$Codebook update step of the MFKM2 algorithm: Calculate ${w_{j}}^{n}=\frac{\sum_{i=1}^{M}\mu_{j}\left(x_{i}\right)x_{i}}{\sum_{i=1}^{M}\mu_{j}\left(x_{i}\right)},\;\forall \;j=1,2,\ldots ,N$
Codebook update with ${w_{j}}^{n+1}={w_{j}}^{n}+s\left(C\left(V\left({w_{j}}^{n}\right)\right)-{w_{j}}^{n}\right)$


## 5. Results

**Figure 1:**
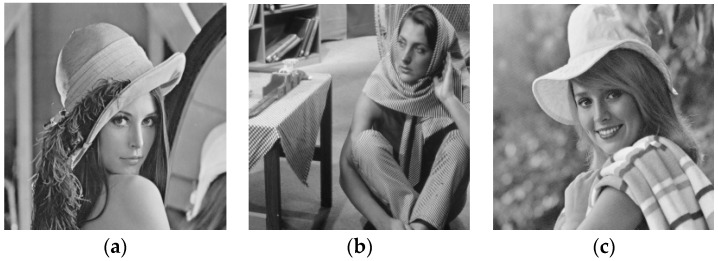

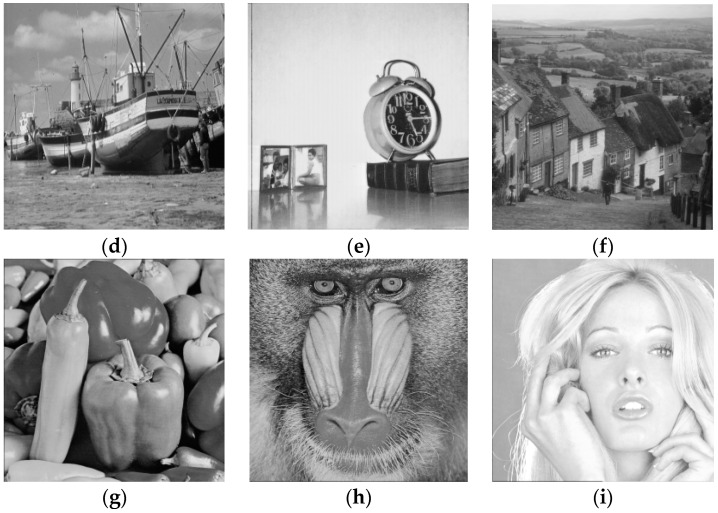
Images 256×256 pixels, 8.0 bpp. (**a**) Lena; (**b**) Barbara; (**c**) Elaine; (**d**) Boat; (**e**) Clock; (**f**) Goldhill; (**g**) Peppers; (**h**) Mandrill; (**i**) Tiffany.

Simulations have been performed in a *core I5*-*2450m* (2.50 GHz) *Intel* computer using nine 256 × 256 pixel images: Lena, Barbara, Elaine, Boat, Clock, Goldhill, Peppers, Mandrill and Tiffany. Each image has 256 gray scale levels, as shown in Figure 1. The parameters used for the simulations were: *K* = 16 (4 × 4 pixel blocks), *N* = 32, 64, 128 and 256, *u =* 2 and two distortion thresholds, $\varepsilon^{\prime }=0.1$ and ε=0.001. For each parameter combination of dimension *K* and codebook size *N* (for example *N* = 32 and *K* = 16), 20 random initializations were used for each algorithm.

Results are presented in terms of average number of iterations and average execution time (in seconds) of the codebook design algorithms, as well as average peak signal noise ratio (PSNR) and structural similarity (SSIM) index [51] of reconstructed images. The notation adopted for the methods are presented in Table 1. Results are organized in Table 2, Table 3, Table 4, Table 5, Table 6, Table 7, Table 8, Table 9, Table 10, Table 11, Table 12, Table 13, Table 14 and Table 15.

Regarding Table 2, all algorithms under consideration led to close values of PSNR. It can be noted that the use of the scale factors led to a decrease in the average number of iterations. In other words, it is observed, for instance, that the average number of iterations of MFKM is smaller than that of FKM. The decrease in the number of iterations is also observed when one compares MFKM1 with FKM1, as well as when one compares MFKM2 with FKM2. The use of PDS for nearest neighbor search contributes to reduce the time spent for codebook design. For instance, considering Elaine image, for FKM1 and FKM1-PDS, the use of PDS in the partitioning step of the second phase (crisp phase) of FKM1 led to a codebook design average time 0.34 s, which is lower than 0.38 s spent for codebook design using the full search (FS) or brute force in that phase. If the ENNS is used in substitution to FS, the time spent is 0.27 s. The highest time savings, concerning FKM1, is obtained by using the scale factor s to decrease the number of iterations combined with the use of ENNS for efficient nearest neighbor search. Indeed, regarding Elaine image, that combination led to an average time spent for codebook design equals 0.25 s.

With respect to Table 3, it is observed that the highest time spent for codebook design was for FKM algorithm. It is important to mention that this behavior is observed for all images and codebook sizes considered in the present work. As an example, for the Boat image and codebook size *N* = 32, the codebook design average time spent by FKM is 1.64 s, which is 8.2 times higher than the average time spent by KM and about 3.8 times higher than the average time spent by FKM2. Table 3 results also confirm the benefits of using the modified versions of the codebook design algorithms (M versions, with the use of the scale factor *s*) and nearest search algorithms for codebook design time savings when compared to the standard versions of the codebook design algorithms. For each image under consideration, it is observed that all algorithms lead to close PSNR values. 

From the results presented in Table 4 and Table 5, it is observed that the codebook design average time spent by FKM2 is higher than that one of FKM1. It is important to mention that the same behavior is observed for all the images under consideration, for codebook sizes 128 and 256. Regarding the number of iterations, it is observed in Table 4 and Table 5 that the modified versions with the use of the scale factor s (algorithms MFKM, MFKM1 and MFKM2) have and average execution time lower than that of the corresponding standard versions (FKM, FKM1 and FKM2 respectively)—due to the savings in the number of iterations. Table 4 and Table 5 point out that the lowest codebook design average time is obtained with the combination of the scale factor s and ENNS. Indeed, considering for instance *fuzzy K*-*means* family 2 and Clock image, in Table 5 the average time of MFKM2-ENNS is 0.92 s, which is lower than the average time presented by all the other versions (FKM2, MFKM2, FKM2-PDS, MFKM2-PDS and FKM2-ENNS).

It is observed in Table 2, Table 3, Table 4 and Table 5 that the best PSNR results, for five out of six images under consideration, for *N* = 32 and *N* = 64, are obtained by using algorithms MFKM2, MFKM2-PDS and MFKM2-ENNS.

From Table 6 and Table 7, for all images under consideration and for all codebook sizes, the modified versions (those using the scale factor *s*) of the algorithms led to average number of iterations smaller than that of the original versions. For instance, for Lena image, the average number of iterations of MFKM is 21.25 and the corresponding number of FKM is 27.60; for Goldhill image, MFKM1 average number of iterations is 16.30 and FKM1 average number of iterations is 20.60; for Boat image, the average number of iterations of MFKM2 is 14.15, and the corresponding number of FKM2 is 16.25. The use of ENNS has proved to be an effective alternative for codebook design time savings. Consider, for instance, Elaine image, for which the codebook design average time of FKM1-ENNS is 0.77 s, while the corresponding time for FKM1 is 1.13 s. For all images under consideration, for each family of *fuzzy K*-*means* algorithm, the highest codebook design time savings is obtained by combining the use of scale factor *s* (M version of the codebook design algorithm) with ENNS. As an example, for all images under consideration, the codebook design average time spent by MFKM2-ENNS is lower than the corresponding one of FKM2, MFKM2, FKM2-PDS, MFKM2-PDS and FKM2-ENNS.

As can be observed in Table 8 and Table 9, in comparison with FKM1 family, the modified version MFKM1 has a smaller average number of iterations, which lead to a lower codebook design average time. Additional time savings is obtained by the use of efficient nearest neighbor search methods, that is, PDS or ENNS. It is important to observe that the modified versions generally lead to higher PSNR values when compared to the original versions. As an example, for Lena image, MFKM1 led to 30.13 dB average PSNR, while the original version led to a corresponding 29.74 dB PSNR; for the same image, the substitution of FKM2 by MFKM2 led to an increase of 0.20 dB in terms of average PSNR.

According to Table 8 and Table 9, for codebook size *N* = 256, for four out of six images under consideration, the best PSNR results are obtained by using algorithms MFKM2, MFKM2-PDS and MFKM2-ENNS. Particularly, for Lena image, the substitution of KM by MFKM2-ENNS lead to a PSNR gain of 0.54 dB.

According to Table 10, the best performance in terms of SSIM is obtained by using MFKM codebooks—the highest SSIM values are observed for MFKM in five out of seven training sets. P-M-T is a training set corresponding to the concatenation of images Peppers, Mandrill and Tiffany. It is important to point out that, for a fixed training set (with the exception of Lena), the absolute difference between the best SSIM result and the worst SSIM result is below 0.0090.

It is observed in Table 11 that MFKM leads to the highest SSIM values for five out of seven training sets. For a fixed training set (with the exception of Elaine and Clock), the absolute difference between the best SSIM result and the worst SSIM result is below 0.0090. 

For *N* = 256, it is observed in Table 13 that MFKM leads to the best SSIM results for 5 out of 7 traning sets considered. An interesting performance nuance must be pointed out—MFKM2, MFKM2-PDS and MFKM2-ENNS are the techniques that lead to the highest PSNR results (according to Table 2, Table 3, Table 4, Table 5, Table 6, Table 7, Table 8 and Table 9), but do not lead to the best SSIM results (as can be observed from Table 10, Table 11, Table 12 and Table 13). It is important to observe that codebook design aims to decrease the distortion (mean square error) obtained in representing the training vectors by the corresponding nearest neighbors, that is, by the corresponding codevectors with minimum distance. In other words, higher PSNR values are obtained by codebooks that are more “tuned” with the training set, that is, by codebooks that introduce less distortion in terms of MSE, which do not necessarily correspond to higher SSIM values. PSNR and SSIM results are presented in Table 14 for images reconstructed by codebooks designed with the training set P-M-T. The method MFKM2-ENNS was used for codebooks designed for *K* = 16 and *N* = 32, 64, 128 and 256, leading to corresponding code rates 0.3125 bpp, 0.375 bpp, 0.4375 bpp and 0.5 bpp. It is observed that, for a given image, both PSNR and SSIM increases with *N*, that is, the distortion decreases with the code rate.

**Figure 2:**
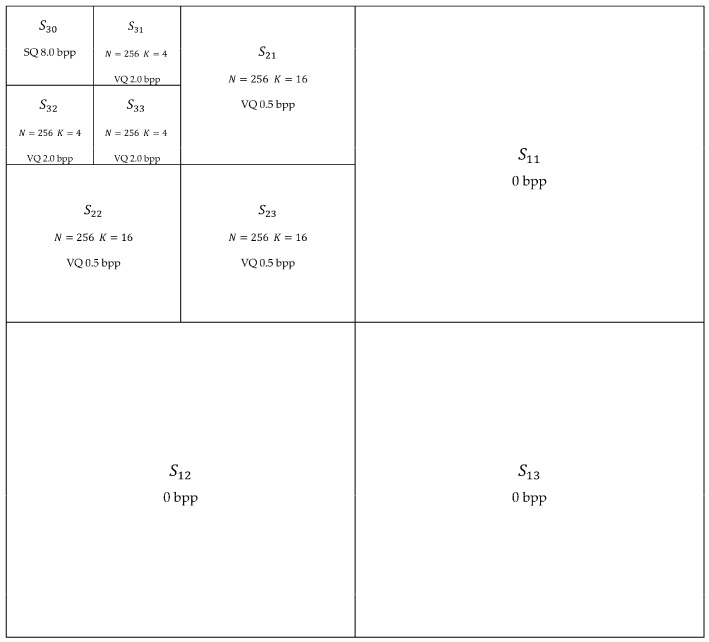
Image encoding using DWT.

The last set of simulations show that vector quantization in the Discrete Wavelet Transform (DWT) domain (that is, by quantizing the wavelet coefficients) lead to reconstructed images with better quality when compared to the ones obtained by VQ in the spatial domain (that is, by quantizing the gray scale values of pixels). For the purpose of DWT VQ [52] at the code rate 0.3125 bpp, a three level multiresolution wavelet decomposition was performed [53] with the wavelet family Daubechies 6. The resulting subbands $S_{ij}$ are submitted to quantization schemes according to Figure 2. 

**Figure 3:**
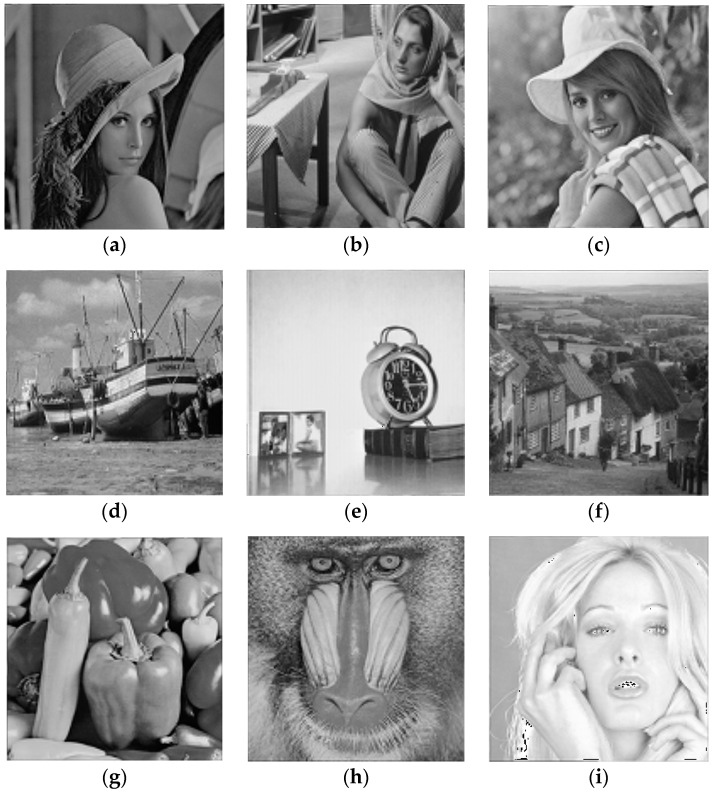
Images obtained from the inverse discrete wavelet transform with the exclusion of subbands *S*_11_, *S*_12_ and *S*_13_. (**a**) Lena PSNR = 30.05 dB; (**b**) Barbara PSNR = 25.54 dB; (**c**) Elaine PSNR = 31.88 dB; (**d**) Boat PSNR = 26.07 dB; (**e**) Clock PSNR = 29.02 dB; (**f**) Goldhill PSNR = 27.77 dB; (**g**) Peppers PSNR = 30.74 dB; (**h**) Mandrill PSNR = 24.93 dB; (**i**) Tiffany PSNR = 31.69 dB.

Subbands $S_{21}$, $S_{22}$ and $S_{23}$ are submitted to the respective wavelet VQ codebooks with *N* = 256 and *K* = 16 (blocks of 4 × 4 wavelet coefficents). Subbands $S_{31}$, $S_{32}$ and $S_{33}$ are submitted to the respective wavelet VQ codebooks with *N* = 256 and *K* = 4 (blocks of 2 × 2 wavelet coefficents). Subband $S_{30}$ is submitted to scalar quantization (SQ) with 8.0 bpp. Subbands $S_{11}$, $S_{12}$ and $S_{13}$ are excluded (that is, code rate 0 bpp)—one can observe in Figure 3 that the application of the inverse discrete wavelet transform after exclusion of subbands $S_{11}$, $S_{12}$ and $S_{13}$, preserving all the other subbands with the wavelet coefficients unchanged, leads to images close to the respective original ones (Figure 1), with good quality, as revealed by visual inspection.

It is worth mentioning that, in the general case, after the application of a multiresolution discrete wavelet transform (DWT) with L resolution levels, the subbands $S_{ij}$, with i=1, 2…, L and j=1, 2, 3, are submitted to multiresolution VQ codebooks. In other words, with the exception of subband $S_{L0}$ (corresponding to the approximation component in the lowest resolution level), each subband is quantized with a specific codebook. The subband $S_{L0}$ is submitted to 8.0 bpp scalar quantization, since it is the subband with the highest importance to the quality of the image obtained from the inverse discrete wavelet transform (IDWT).

Assume the general case of an image with P × P pixels. The number of wavelet coefficients in $S_{ij}$, with 1≤i≤L, is $\frac{P\;\times \;P}{2^{i}\;\times \;2^{i}}$. Let $R_{S_{ij}}$ be the code rate (in bpp or, correspondingly, in bit/coefficient) of VQ for subband $S_{ij}$, 1≤i≤L and 1≤j≤3, and $R_{S_{L0}}$ be the code rate (in bpp) of scalar quantization for subband $S_{L0}$. The final code rate $R_{T}$ (in bpp) of the image coding using DWT (with L resolution levels) and VQ is given by:

(16) $$R_{T}=\;\frac{1}{P\;\times \;P}\;\left(\frac{P\;\times \;P}{2^{L}\;\times \;2^{L}}R_{S_{L0}\;}+\;\sum_{i=1}^{L}\sum_{j=1}^{3}\frac{P\;\times \;P}{2^{i}\;\times \;2^{i}}\;R_{S_{ij}\;}\right),$$

that is:

(17) $$R_{T}=\;\frac{R_{S_{L0}\;}}{2^{2L}}+\sum_{i=1}^{L}\sum_{j=1}^{3}\frac{R_{S_{ij}\;}}{2^{2i}}.$$

For VQ with dimension K and codebook size N, it follows that the corresponding code rate is $\frac{1}{K}\;\log_{2}N$. Hence, according to Figure 2, it follows that:

(18) $$R_{S_{21}\;}=\;R_{S_{22}\;}=\;R_{S_{23}\;}=\;\frac{1}{16}\log_{2}256=0.5\;\mathrm{bpp}$$

and:

(19) $$R_{S_{31}\;}=\;R_{S_{32}\;}=\;R_{S_{33}\;}=\;\frac{1}{4}\log_{2}256=2.0\;\mathrm{bpp}.$$

From Figure 2, it follows that $R_{S_{30}\;}=8.0\;\mathrm{bpp}$ and $R_{S_{11}\;}=\;R_{S_{12}\;}=\;R_{S_{13}\;}=0\;\mathrm{bpp}$. Thus, from Equation (17), the corresponding overall code rate under the conditions presented in Figure 2 is $R_{T}=0.3125\;\mathrm{bpp}.$ It is worth mentioning that the importance of subbands $S_{ij}$ for the image quality increases with i—that is the reason why $R_{S_{3j}\;}>\;R_{S_{2j}}$, for j=1, 2, 3. 

**Figure 4:**
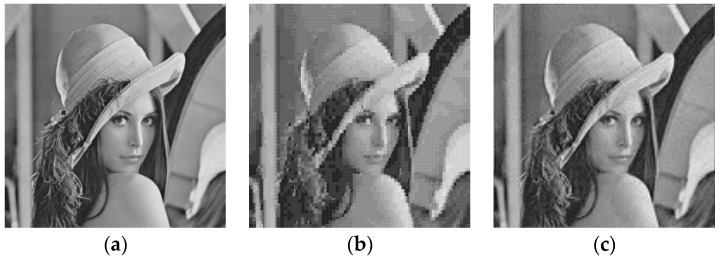
Images Lena: (**a**) Original; (**b**) Reconstructed using spatial domain VQ with 0.3125 bpp (PSNR = 25.62 dB and SSIM = 0.7211); (**c**) Reconstructed using DWT VQ with 0.3125 bpp (PSNR = 29.35 dB and SSIM = 0.8367). Codebooks were designed with training set P-M-T by MFKM2-ENNS.

**Figure 5:**
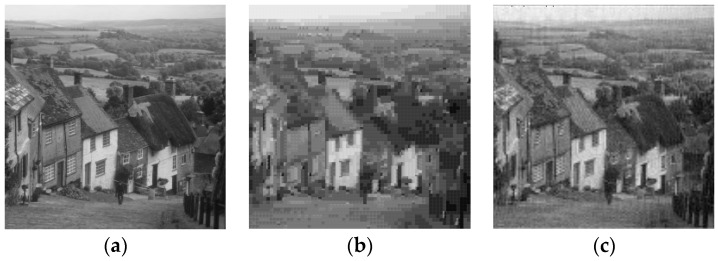
Images Goldhill: (**a**) Original; (**b**) Reconstructed using spatial domain VQ with 0.3125 bpp (PSNR = 25.71 dB and SSIM = 0.6391); (**c**) Reconstructed using DWT VQ with 0.3125 bpp (PSNR = 26.81 dB and SSIM = 0.7640). Codebooks were designed with training set P-M-T by MFKM2-ENNS.

As can be observed in Figure 4 and Figure 5, visual inspections of the reconstructed images reveal the superiority of DWT VQ over vector quantization in the spatial domain. The superiority is also confirmed in terms of PSNR and SSIM values. 

The superiority of DWT VQ over spatial domain VQ is also observed in Table 15. As an example, by using P-M-T as the training set, PSNR gain of 3.10 dB for Elaine image is obtained by substituting spatial domain VQ by DWT VQ. For a given image, one can observe that better PSNR and SSIM results are obtained by DWT VQ with codebooks designed by P-M-T when compared to spatial domain VQ with codebook designed by the image itself. Consider, for instance, the Lena image. If the Lena image is reconstructed using spatial domain VQ with codebook designed by itself as training set, a PSNR 26.72 dB and a SSIM 0.7791 are obtained. If the Lena image is reconstructed in the DWT domain with multiresolution codebooks designed by P-M-T as training set, a PSNR 29.35 dB and a SSIM 0.8367 are obtained.

As a final comment, image coding based on VQ is one of the possible applications of the families of *fuzzy K*-*means* algorithms considered in this paper. The focus of the present work is to assess the fact that the proposed acceleration techniques make VQ codebook design faster, since other efficient image coding techniques exist.

## 6. Conclusions 

In this work, alternatives were presented for accelerating families of *fuzzy K*-*means* algorithms applied to vector quantization codebook design. A lookahead approach was used with the purpose of decreasing the number of iterations of the algorithms. The approach consists in using a scale factor in the computation of the codevectors.

An additional acceleration was obtained by accommodating efficient nearest neighbor search techniques in the partitioning step of the algorithms. With such approach, savings are obtained in the number of operations spent by the algorithms. The combination of the scale factor (lookahead approach) with efficient nearest neighbor search was evaluated in the scenario of image vector quantization codebook design. Savings up to 40% in the time spent for codebook design were obtained, without sacrificing the quality of the codebook, assessed by the peak signal-to-noise ratio (PSNR) as well as by structural similarity (SSIM) index of the reconstructed images. 

## Figures and Tables

**Table 1:** Notation.

KM	*K-means*
FKM	*Fuzzy K-means*
MFKM	*Modified Fuzzy K-means* (accelerated version with scale s)
FKM1	*Fuzzy K-means Family* 1
MFKM1	*Modified Fuzzy K-means Family* 1 (accelerated version with scale s)
FKM1-PDS	*Fuzzy K-means Family* 1 with Partial Distortion Search in the crisp phase
MFKM1-PDS	*Modified Fuzzy K-means Family* 1 (accelerated version with scale s) with Partial Distortion Search in the crisp phase
FKM1-ENNS	*Fuzzy K-means Family* 1 with Equal-Average Nearest Neighbor Search in the crisp phase
MFKM1-ENNS	*Modified Fuzzy K-means Family* 1 (accelerated version with scale s) with Equal-Average Nearest Neighbor Search in the crisp phase
FKM2	*Fuzzy K-means Family* 2
MFKM2	*Modified Fuzzy K-means Family* 2 (accelerated version with scale s)
FKM2-PDS	*Fuzzy K-means Family* 2 with Partial Distortion Search in the crisp phase
MFKM2-PDS	*Modified Fuzzy K-means Family* 2 (accelerated version with scale s) with Partial Distortion Search in the crisp phase
FKM2-ENNS	*Fuzzy K-means Family* 2 with Equal-Average Nearest Neighbor Search in the crisp phase
MFKM2-ENNS	*Modified Fuzzy K-means Family* 2 (accelerated version with scale s) with Equal-Average Nearest Neighbor Search in the crisp phase

**Table 2:** PSNR (in dB), number of iterations and codebook design time (in seconds) for images Lena, Barbara and Elaine, using *N* = 32.

Algorithm	Lena			Barbara			Elaine		
	PSNR	Iter	Time	PSNR	Iter	Time	PSNR	Iter	Time
KM	26.61	17.20	0.16	24.76	15.20	0.12	27.75	18.15	0.16
FKM	26.57	19.65	1.53	24.71	16.00	1.11	27.70	19.75	1.65
MFKM	26.61	14.75	1.12	24.72	12.30	0.86	27.72	15.80	1.31
FKM1	26.60	22.35	0.35	24.77	19.00	0.38	27.77	24.35	0.38
MFKM1	26.62	18.25	0.28	24.79	16.05	0.31	27.77	18.55	0.30
FKM1-PDS	26.60	22.35	0.33	24.77	19.00	0.34	27.77	24.35	0.34
MFKM1-PDS	26.62	18.25	0.26	24.79	16.05	0.28	27.77	18.55	0.29
FKM1-ENNS	26.60	22.35	0.26	24.77	19.00	0.28	27.77	24.35	0.27
MFKM1-ENNS	26.62	18.25	0.22	24.79	16.05	0.25	27.77	18.55	0.25
FKM2	26.60	15.35	0.39	24.77	14.25	0.30	27.77	18.50	0.40
MFKM2	26.63	12.70	0.35	24.78	11.75	0.24	27.80	14.40	0.33
FKM2-PDS	26.60	15.35	0.35	24.77	14.25	0.27	27.77	18.45	0.37
MFKM2-PDS	26.63	12.70	0.33	24.78	11.75	0.22	27.80	14.40	0.30
FKM2-ENNS	26.60	15.35	0.33	24.77	14.25	0.24	27.77	18.50	0.29
MFKM2-ENNS	26.63	12.70	0.30	24.78	11.75	0.20	27.80	14.40	0.26

**Table 3:** PSNR (in dB), number of iterations and codebook design time (in seconds) for images Boat, Clock and Goldhill, using *N* = 32.

Algorithm	Boat			Clock			Goldhill		
	PSNR	Iter	Time	PSNR	Iter	Time	PSNR	Iter	Time
KM	24.92	18.95	0.20	26.16	26.10	0.32	26.66	17.00	0.34
FKM	24.84	21.20	1.64	26.23	41.00	1.78	26.67	18.60	3.05
MFKM	24.87	16.20	0.98	26.28	35.55	1.08	26.70	16.30	2.57
FKM1	24.91	25.75	0.42	26.19	33.40	0.55	26.67	22.25	0.46
MFKM1	24.93	19.80	0.33	26.25	25.80	0.45	26.68	18.85	0.36
FKM1-PDS	24.91	25.75	0.40	26.19	33.40	0.50	26.67	22.25	0.43
MFKM1-PDS	24.93	19.80	0.31	26.25	25.80	0.41	26.68	18.85	0.32
FKM1-ENNS	24.91	25.75	0.32	26.19	33.40	0.39	26.67	22.25	0.35
MFKM1-ENNS	24.93	19.80	0.27	26.25	25.80	0.34	26.68	18.85	0.30
FKM2	24.91	18.90	0.43	26.26	23.30	0.46	26.67	16.20	0.63
MFKM2	24.93	15.20	0.37	26.32	20.50	0.40	26.70	13.55	0.55
FKM2-PDS	24.91	18.90	0.41	26.26	23.30	0.43	26.67	16.20	0.58
MFKM2-PDS	24.93	15.20	0.35	26.32	20.50	0.39	26.70	13.55	0.51
FKM2-ENNS	24.91	18.90	0.36	26.26	23.30	0.40	26.67	16.20	0.47
MFKM2-ENNS	24.93	15.20	0.32	26.32	20.50	0.34	26.70	13.55	0.46

**Table 4:** PSNR (in dB), number of iterations and codebook design time (in seconds) for images Lena, Barbara and Elaine, using *N* = 64.

Algorithm	Lena			Barbara			Elaine		
	PSNR	Iter	Time	PSNR	Iter	Time	PSNR	Iter	Time
KM	27.74	17.80	0.32	25.68	16.25	0.25	29.06	18.00	0.24
FKM	27.69	22.85	5.81	25.64	21.15	4.03	29.09	24.05	4.91
MFKM	27.73	17.90	4.51	25.64	15.40	3.31	29.13	18.60	3.98
FKM1	27.67	23.15	0.61	25.74	20.65	0.55	29.01	23.55	0.62
MFKM1	27.75	19.10	0.48	25.77	17.95	0.48	29.07	19.90	0.53
FKM1-PDS	27.67	23.15	0.53	25.74	20.65	0.51	29.01	23.55	0.54
MFKM1-PDS	27.75	19.10	0.46	25.77	17.95	0.44	29.07	19.90	0.47
FKM1-ENNS	27.67	23.15	0.39	25.74	20.65	0.43	29.01	23.55	0.46
MFKM1-ENNS	27.75	19.10	0.35	25.77	17.95	0.36	29.07	19.90	0.40
FKM2	27.80	14.50	0.81	25.73	15.00	0.71	29.05	16.45	0.81
MFKM2	27.85	12.85	0.71	25.75	12.85	0.61	29.10	13.70	0.75
FKM2-PDS	27.80	14.50	0.70	25.73	15.00	0.62	29.05	16.45	0.74
MFKM2-PDS	27.85	12.85	0.67	25.75	12.85	0.55	29.10	13.70	0.68
FKM2-ENNS	27.80	14.50	0.62	25.73	14.95	0.57	29.05	16.40	0.62
MFKM2-ENNS	27.85	12.85	0.60	25.75	12.85	0.52	29.10	13.70	0.60

**Table 5:** PSNR (in dB), number of iterations and codebook design time (in seconds) for images Boat, Clock and Goldhill, using *N* = 64.

Algorithm	Boat			Clock			Goldhill		
	PSNR	Iter	Time	PSNR	Iter	Time	PSNR	Iter	Time
KM	25.90	18.45	0.46	27.17	22.05	0.62	27.69	16.15	0.36
FKM	25.84	23.30	6.31	27.41	42.70	8.11	27.68	19.55	5.81
MFKM	25.85	16.05	4.61	27.46	33.85	5.53	27.70	15.20	4.51
FKM1	25.85	24.35	0.73	27.08	25.10	1.05	27.69	21.80	0.64
MFKM1	25.91	18.90	0.62	27.16	20.10	0.80	27.71	18.05	0.53
FKM1-PDS	25.85	24.35	0.68	27.08	25.65	0.88	27.69	21.85	0.60
MFKM1-PDS	25.91	18.90	0.56	27.16	20.10	0.70	27.71	18.05	0.49
FKM1-ENNS	25.85	24.35	0.55	27.08	25.10	0.68	27.69	21.80	0.48
MFKM1-ENNS	25.91	18.90	0.44	27.16	20.10	0.52	27.71	18.05	0.43
FKM2	25.92	17.50	0.94	27.32	18.90	1.09	27.70	15.60	1.04
MFKM2	25.96	13.80	0.85	27.40	16.10	1.01	27.73	13.10	0.89
FKM2-PDS	25.92	17.45	0.87	27.32	18.90	1.03	27.70	15.55	0.96
MFKM2-PDS	25.96	13.80	0.83	27.40	16.10	0.98	27.73	13.10	0.93
FKM2-ENNS	25.92	17.50	0.84	27.32	18.90	0.94	27.70	15.60	0.83
MFKM2-ENNS	25.96	13.80	0.70	27.40	16.10	0.92	27.73	13.10	0.75

**Table 6:** PSNR (in dB), number of iterations and codebook design time (in seconds) for images Lena, Barbara and Elaine, using *N* = 128.

Algorithm	Lena			Barbara			Elaine		
	PSNR	Iter	Time	PSNR	Iter	Time	PSNR	Iter	Time
KM	28.83	18.10	0.51	26.68	14.95	0.45	30.27	16.30	0.51
FKM	28.91	27.60	22.31	26.61	20.35	13.45	30.40	26.15	18.47
MFKM	28.95	21.25	16.32	26.64	16.70	11.57	30.44	19.50	13.11
FKM1	28.73	22.20	1.10	26.74	20.80	1.06	30.17	23.10	1.13
MFKM1	28.92	17.55	0.91	26.81	16.05	0.85	30.30	18.55	0.91
FKM1-PDS	28.73	22.25	0.96	26.74	20.90	0.96	30.17	23.10	1.05
MFKM1-PDS	28.92	17.55	0.81	26.81	15.95	0.75	30.30	18.55	0.82
FKM1-ENNS	28.73	22.20	0.79	26.74	20.80	0.77	30.17	23.10	0.77
MFKM1-ENNS	28.92	17.55	0.68	26.81	16.05	0.63	30.30	18.55	0.68
FKM2	28.97	14.45	1.97	26.74	14.30	1.60	30.34	14.35	1.83
MFKM2	29.07	12.55	1.76	26.79	12.85	1.54	30.45	12.70	1.73
FKM2-PDS	28.97	14.45	1.76	26.74	14.30	1.55	30.34	14.35	1.72
MFKM2-PDS	29.07	12.55	1.65	26.79	12.85	1.48	30.45	12.70	1.66
FKM2-ENNS	28.97	14.45	1.60	26.74	14.30	1.47	30.34	14.30	1.59
MFKM2-ENNS	29.07	12.55	1.56	26.79	12.85	1.39	30.45	12.70	1.57

**Table 7:** PSNR (in dB), number of iterations and codebook design time (in seconds) for images Boat, Clock and Goldhill, using *N* = 128.

Algorithm	Boat			Clock			Goldhill		
	PSNR	Iter	Time	PSNR	Iter	Time	PSNR	Iter	Time
KM	26.90	17.80	0.53	28.28	16.60	0.65	28.67	15.05	0.41
FKM	26.91	26.85	25.38	28.48	31.40	39.61	28.66	20.30	13.51
MFKM	26.94	20.70	22.56	28.55	26.05	36.47	28.69	15.35	11.02
FKM1	26.59	24.15	1.22	28.04	20.50	1.36	28.69	20.60	1.15
MFKM1	26.70	17.20	0.98	28.24	17.50	1.13	28.77	16.30	0.99
FKM1-PDS	26.59	24.15	1.17	28.04	20.35	1.26	28.69	20.75	1.02
MFKM1-PDS	26.70	17.20	0.93	28.24	17.50	1.03	28.77	16.30	0.95
FKM1-ENNS	26.59	24.15	0.90	28.04	20.50	0.85	28.69	20.60	0.90
MFKM1-ENNS	26.70	17.20	0.72	28.24	17.50	0.73	28.77	16.30	0.83
FKM2	26.97	16.25	2.04	28.28	14.40	2.56	28.69	14.30	1.80
MFKM2	27.07	14.15	1.85	28.40	13.15	2.48	28.75	12.95	1.75
FKM2-PDS	26.97	16.25	1.97	28.28	14.40	2.47	28.69	14.30	1.76
MFKM2-PDS	27.07	14.15	1.76	28.40	13.15	2.45	28.75	12.95	1.70
FKM2-ENNS	26.97	16.25	1.77	28.28	14.40	2.32	28.69	14.35	1.64
MFKM2-ENNS	27.07	14.15	1.68	28.40	13.15	2.30	28.75	12.95	1.52

**Table 8:** PSNR (in dB), number of iterations and codebook design time (in seconds) for images Lena, Barbara and Elaine, using *N* = 256.

Algorithm	Lena			Barbara			Elaine		
	PSNR	Iter	Time	PSNR	Iter	Time	PSNR	Iter	Time
KM	29.89	14.70	0.62	27.76	13.60	0.58	31.46	14.40	0.66
FKM	30.21	38.20	90.19	27.74	25.25	66.32	31.80	31.65	79.91
MFKM	30.24	27.10	73.28	27.76	20.00	57.34	31.87	24.30	75.85
FKM1	29.74	21.80	1.99	27.78	18.40	1.89	31.16	20.65	1.97
MFKM1	30.13	16.20	1.78	27.97	15.15	1.71	31.53	17.10	1.75
FKM1-PDS	29.74	21.80	1.78	27.78	18.40	1.74	31.16	20.65	1.73
MFKM1-PDS	30.13	16.20	1.56	27.97	15.15	1.59	31.53	17.10	1.52
FKM1-ENNS	29.74	21.75	1.40	27.78	18.50	1.41	31.16	20.75	1.46
MFKM1-ENNS	30.13	16.20	1.36	27.97	15.15	1.40	31.53	17.10	1.38
FKM2	30.23	14.10	5.04	27.88	13.35	5.10	31.65	13.10	5.32
MFKM2	30.43	12.75	5.17	28.00	12.05	5.04	31.77	12.25	5.79
FKM2-PDS	30.23	14.10	4.92	27.88	13.35	4.81	31.65	13.10	5.16
MFKM2-PDS	30.43	12.75	5.22	28.00	12.05	4.65	31.77	12.25	5.42
FKM2-ENNS	30.23	14.10	4.63	27.88	13.30	4.65	31.65	13.10	4.92
MFKM2-ENNS	30.43	12.75	4.94	28.00	12.05	4.58	31.77	12.25	5.37

**Table 9:** PSNR (in dB), number of iterations and codebook design time (in seconds) for images Boat, Clock and Goldhill, using *N* = 256.

Algorithm	Boat			Clock			Goldhill		
	PSNR	Iter	Time	PSNR	Iter	Time	PSNR	Iter	Time
KM	27.91	13.30	0.63	29.47	13.70	0.65	29.73	13.30	0.61
FKM	28.04	32.05	84.26	29.82	35.65	81.15	29.83	24.70	63.14
MFKM	28.08	23.65	70.18	29.85	26.00	75.34	29.86	18.50	54.12
FKM1	27.57	24.05	2.44	29.09	19.75	1.81	29.68	19.30	2.12
MFKM1	27.87	17.55	1.93	29.41	16.10	1.59	29.90	15.80	1.88
FKM1-PDS	27.57	24.05	2.25	29.09	19.75	1.64	29.68	19.30	1.96
MFKM1-PDS	27.87	17.55	1.79	29.41	16.10	1.42	29.90	15.80	1.76
FKM1-ENNS	27.57	23.95	1.77	29.09	19.70	1.29	29.68	19.35	1.51
MFKM1-ENNS	27.87	17.55	1.43	29.41	16.10	1.12	29.90	15.80	1.42
FKM2	28.05	13.40	5.29	29.56	12.75	5.10	29.80	12.10	5.12
MFKM2	28.22	11.85	5.53	29.75	12.40	5.35	29.92	11.80	5.62
FKM2-PDS	28.05	13.40	5.09	29.56	12.75	4.82	29.80	12.10	5.05
MFKM2-PDS	28.22	11.85	5.15	29.75	12.40	5.12	29.92	11.80	5.34
FKM2-ENNS	28.05	13.40	4.76	29.56	12.75	4.52	29.80	12.10	4.83
MFKM2-ENNS	28.22	11.85	5.06	29.75	12.40	4.50	29.92	11.80	5.21

**Table 10:** SSIM for images Lena, Barbara Elaine, Boat, Clock, Goldhill and P-M-T, using *N* = 32.

Algorithm	SSIM						
	Lena	Barbara	Elaine	Boat	Clock	Goldhill	P-M-T
KM	0.7790	0.6800	0.7637	0.7081	0.8373	0.7078	0.7492
FKM	0.7838	0.6809	0.7687	0.7118	0.8447	0.7105	0.7501
MFKM	0.7840	0.6807	0.7688	0.7120	0.8457	0.7111	0.7496
FKM1	0.7816	0.6807	0.7678	0.7109	0.8381	0.7105	0.7481
MFKM1	0.7813	0.6813	0.7674	0.7095	0.8386	0.7110	0.7502
FKM1-PDS	0.7816	0.6807	0.7678	0.7109	0.8381	0.7105	0.7481
MFKM1-PDS	0.7813	0.6813	0.7674	0.7095	0.8386	0.7110	0.7502
FKM1-ENNS	0.7816	0.6807	0.7678	0.7109	0.8381	0.7105	0.7481
MFKM1-ENNS	0.7813	0.6813	0.7674	0.7095	0.8386	0.7110	0.7502
FKM2	0.7731	0.6787	0.7617	0.7083	0.8383	0.7083	0.7483
MFKM2	0.7736	0.6793	0.7622	0.7075	0.8395	0.7087	0.7490
FKM2-PDS	0.7731	0.6787	0.7617	0.7083	0.8383	0.7083	0.7483
MFKM2-PDS	0.7736	0.6793	0.7622	0.7075	0.8395	0.7087	0.7490
FKM2-ENNS	0.7731	0.6787	0.7617	0.7083	0.8383	0.7083	0.7483
MFKM2-ENNS	0.7736	0.6793	0.7622	0.7075	0.8395	0.7087	0.7490

**Table 11:** SSIM for images Lena, Barbara Elaine, Boat, Clock, Goldhill and P-M-T, using *N* = 64.

Algorithm	SSIM						
	Lena	Barbara	Elaine	Boat	Clock	Goldhill	P-M-T
KM	0.8225	0.7323	0.8094	0.7652	0.8667	0.7613	0.7897
FKM	0.8260	0.7325	0.8136	0.7657	0.8749	0.7628	0.7902
MFKM	0.8261	0.7318	0.8137	0.7653	0.8756	0.7631	0.7910
FKM1	0.8228	0.7355	0.8105	0.7655	0.8656	0.7629	0.7900
MFKM1	0.8224	0.7351	0.8096	0.7643	0.8661	0.7622	0.7902
FKM1-PDS	0.8228	0.7355	0.8105	0.7655	0.8657	0.7629	0.7900
MFKM1-PDS	0.8224	0.7351	0.8096	0.7643	0.8661	0.7622	0.7902
FKM1-ENNS	0.8228	0.7355	0.8105	0.7655	0.8656	0.7629	0.7900
MFKM1-ENNS	0.8224	0.7351	0.8096	0.7643	0.8661	0.7622	0.7902
FKM2	0.8177	0.7312	0.8031	0.7646	0.8680	0.7605	0.7900
MFKM2	0.8179	0.7316	0.8032	0.7645	0.8692	0.7610	0.7897
FKM2-PDS	0.8177	0.7312	0.8031	0.7646	0.8680	0.7604	0.7900
MFKM2-PDS	0.8179	0.7316	0.8032	0.7645	0.8692	0.7610	0.7897
FKM2-ENNS	0.8177	0.7312	0.8031	0.7646	0.8680	0.7605	0.7900
MFKM2-ENNS	0.8179	0.7316	0.8032	0.7645	0.8692	0.7610	0.7897

**Table 12:** SSIM for images Lena, Barbara Elaine, Boat, Clock, Goldhill and P-M-T, using *N* = 128.

Algorithm	SSIM						
	Lena	Barbara	Elaine	Boat	Clock	Goldhill	P-M-T
KM	0.8583	0.7863	0.8487	0.8141	0.8941	0.8050	0.8232
FKM	0.8617	0.7864	0.8523	0.8143	0.9006	0.8066	0.8219
MFKM	0.8616	0.7864	0.8523	0.8138	0.9014	0.8063	0.8224
FKM1	0.8579	0.7881	0.8481	0.8035	0.8876	0.8076	0.8231
MFKM1	0.8575	0.7855	0.8475	0.8024	0.8889	0.8062	0.8233
FKM1-PDS	0.8579	0.7881	0.8481	0.8035	0.8876	0.8076	0.8231
MFKM1-PDS	0.8575	0.7856	0.8475	0.8024	0.8889	0.8062	0.8233
FKM1-ENNS	0.8579	0.7881	0.8481	0.8035	0.8876	0.8076	0.8231
MFKM1-ENNS	0.8575	0.7855	0.8475	0.8024	0.8889	0.8062	0.8233
FKM2	0.8518	0.7823	0.8387	0.8124	0.8899	0.8041	0.8236
MFKM2	0.8520	0.7833	0.8394	0.8128	0.8906	0.8048	0.8244
FKM2-PDS	0.8518	0.7823	0.8387	0.8124	0.8899	0.8041	0.8236
MFKM2-PDS	0.8520	0.7833	0.8394	0.8128	0.8906	0.8048	0.8244
FKM2-ENNS	0.8518	0.7823	0.8387	0.8124	0.8899	0.8041	0.8236
MFKM2-ENNS	0.8520	0.7833	0.8394	0.8128	0.8906	0.8048	0.8244

**Table 13:** SSIM for images Lena, Barbara Elaine, Boat, Clock, Goldhill and P-M-T, using *N* = 256.

Algorithm	SSIM						
	Lena	Barbara	Elaine	Boat	Clock	Goldhill	P-M-T
KM	0.8893	0.8351	0.8808	0.8514	0.9173	0.8450	0.8534
FKM	0.8935	0.8371	0.8843	0.8540	0.9226	0.8478	0.8516
MFKM	0.8935	0.8372	0.8843	0.8539	0.9231	0.8479	0.8518
FKM1	0.8875	0.8349	0.8764	0.8478	0.9095	0.8452	0.8530
MFKM1	0.8877	0.8322	0.8761	0.8490	0.9100	0.8442	0.8529
FKM1-PDS	0.8875	0.8349	0.8764	0.8478	0.9095	0.8452	0.8530
MFKM1-PDS	0.8877	0.8322	0.8761	0.8490	0.9100	0.8442	0.8529
FKM1-ENNS	0.8875	0.8349	0.8764	0.8478	0.9096	0.8452	0.8530
MFKM1-ENNS	0.8877	0.8322	0.8761	0.8490	0.9100	0.8442	0.8529
FKM2	0.8842	0.8333	0.8690	0.8520	0.9145	0.8450	0.8550
MFKM2	0.8852	0.8339	0.8696	0.8527	0.9155	0.8460	0.8553
FKM2-PDS	0.8842	0.8333	0.8690	0.8520	0.9145	0.8450	0.8550
MFKM2-PDS	0.8852	0.8339	0.8696	0.8527	0.9155	0.8460	0.8553
FKM2-ENNS	0.8842	0.8333	0.8690	0.8519	0.9145	0.8450	0.8550
MFKM2-ENNS	0.8852	0.8339	0.8696	0.8527	0.9155	0.8460	0.8553

**Table 14:** PSNR (in dB) and SSIM of reconstructed images by using codebooks designed with the training set P-M-T, using MFKM2-ENNS.

Images	*N* = 32		*N* = 64		*N* = 128		*N* = 256	
	PSNR	SSIM	PSNR	SSIM	PSNR	SSIM	PSNR	SSIM
Lena	25.62	0.7211	26.34	0.7604	26.91	0.7816	27.50	0.8133
Barbara	24.09	0.6350	24.66	0.6679	25.19	0.6982	25.68	0.7293
Elaine	26.62	0.7223	27.51	0.7626	28.11	0.7848	28.88	0.8134
Boat	24.16	0.6575	24.88	0.7038	25.31	0.7259	25.89	0.7633
Clock	25.21	0.7991	26.05	0.8207	26.81	0.8470	27.32	0.8618
Goldhill	25.71	0.6391	26.34	0.6788	26.92	0.7132	27.45	0.7435
Tiffany	28.21	0.7493	29.10	0.7917	30.40	0.8365	31.38	0.8647

**Table 15:** PSNR (in dB) and SSIM of reconstructed images. Codebooks were designed using MFKM2-ENNS in spatial domain as well as by the DWT domain for code rate 0.3125 bpp.

Images	Spatial Domain VQ. Performance Inside the Training Set		Spatial Domain VQ with Codebooks Designed by Using P-M-T Training Set		DWT VQ with Multiresolution Codebooks Designed by Using P-M-T Training Set	
	PSNR	SSIM	PSNR	SSIM	PSNR	SSIM
Lena	26.72	0.7791	25.62	0.7211	29.35	0.8367
Barbara	24.78	0.6822	24.09	0.6350	25.00	0.7573
Elaine	27.79	0.7566	26.62	0.7223	29.72	0.8304
Boat	24.90	0.7047	24.16	0.6575	25.49	0.7581
Clock	26.27	0.8364	25.21	0.7991	28.22	0.8672
Goldhill	26.76	0.7085	25.71	0.6391	26.81	0.7640
Tiffany	29.01	0.8078	28.21	0.7493	30.21	0.8099
